# The Sickle Cell Disease Ontology: Enabling Collaborative Research and Co-Designing of New Planetary Health Applications

**DOI:** 10.1089/omi.2020.0153

**Published:** 2020-10-05

**Authors:** Victoria Nembaware, Gaston K. Mazandu, Jade Hotchkiss, Jean-Michel Safari Serufuri, Jill Kent, Andre Pascal Kengne, Kofi Anie, Nchangwi Syntia Munung, Daima Bukini, Valentina Josiane Ngo Bitoungui, Deogratias Munube, Uzima Chirwa, Catherine Chunda-Liyoka, Agnes Jonathan, Miriam V. Flor-Park, Kevin Kum Esoh, Mario Jonas, Khuthala Mnika, Chandré Oosterwyk, Upendo Masamu, Jack Morrice, Annette Uwineza, Arthemon Nguweneza, Kambe Banda, Isaac Nyanor, David Nana Adjei, Nathan Edward Siebu, Malula Nkanyemka, Patience Kuona, Bamidele O. Tayo, Andrew Campbell, Assaf P. Oron, Obiageli E. Nnodu, Vivian Painstil, Julie Makani, Nicola Mulder, Ambroise Wonkam

**Affiliations:** ^1^ Division of Human Genetics, Faculty of Health Sciences, University of Cape Town, Cape Town, South Africa.; ^2^ Sickle Cell Programme, Muhimbili University of Health and Allied Sciences (MUHAS), Dar es Salaam, Tanzania.; ^3^ Non-Communicable Diseases Research Unit, South African Medical Research Council, Cape Town, South Africa.; ^4^ London North West University Healthcare NHS Trust and Imperial College London, London, UK.; ^5^ Sickle Cell Disease Genomics Network of Africa (SickleGenAfrica), University of Ghana, Accra, Ghana.; ^6^ Department of Microbiology, Hematology and Immunology, Faculty of Medicine and Pharmaceutical Sciences of the University of Dschang, Dschang, Cameroon.; ^7^ Department of Paediatric and Child Health, Makerere University/Mulago National Referral Hospital, Kampala, Uganda.; ^8^ University Teaching Hospitals—Children's Hospital, University of Zambia, School of Medicine, Lusaka, Zambia.; ^9^ Onco-hematology Unit, Instituto da Criança, Hospital das Clínicas, Universidade de São Paulo, São Paulo, Brazil.; ^10^ Department of Biochemistry, Faculty of Science, Jomo Kenyatta University of Agriculture and Technology, Juja, Kenya.; ^11^ University of Rwanda, School of Medicine and Pharmacy, Kigali, Rwanda.; ^12^ Kumasi Centre for Sickle Cell Disease, Komfo Anokye Teaching Hospital, Accra, Ghana.; ^13^ University of Zimbabwe College of Health Sciences, Harare, Zimbabwe.; ^14^ Department of Public Health Sciences, Parkinson School of Health Sciences and Public Health, Loyola University Chicago, Maywood, Illinois, USA.; ^15^ Division of Hematology, Center for Cancer and Blood Disorders, Children's National Medical Center, George Washington University School of Medicine and Health Sciences, Washington, DC, USA.; ^16^ Maternal, Newborn and Child Health, Institute for Disease Modeling, Bellevue, Washington, USA.; ^17^ Centre of Excellence for Sickle Cell Disease Research and Training, University of Abuja, Abuja, Nigeria.; ^18^ Department of Child Health, Komfo Anokye Teaching Hospital, Kumasi, Ghana.; ^19^ Computational Biology Division, Faculty of Health Sciences, Cape Town, South Africa.

**Keywords:** sickle cell disease, sickle cell disease ontology, SickleInAfrica, Sickle Africa Data Coordinating Center, planetary health, data harmonization, Global Clinical Trial Design

## Abstract

Sickle cell disease (SCD) is one of the most common blood disorders impacting planetary health. Over 300,000 newborns are diagnosed with SCD each year globally, with an increasing trend. The sickle cell disease ontology (SCDO) is the most comprehensive multidisciplinary SCD knowledge portal. The SCDO was collaboratively developed by the SCDO working group, which includes experts in SCD and data standards from across the globe. This expert review presents highlights and lessons learned from the fourth SCDO workshop that marked the beginning of applications toward planetary health impact, and with an eye to empower and cultivate multisite SCD collaborative research. The workshop was organized by the Sickle Africa Data Coordinating Center (SADaCC) and attended by 44 participants from 14 countries, with 2 participants connecting remotely. Notably, from the standpoint of democratizing and innovating scientific meeting design, an SCD patient advocate also presented at the workshop, giving a broader real-life perspective on patients' aspirations, needs, and challenges. A major component of the workshop was new approaches to harness SCDO to harmonize data elements used by different studies. This was facilitated by a web-based platform onto which participants uploaded data elements from previous or ongoing SCD-relevant research studies before the workshop, making multisite collaborative research studies based on existing SCD data possible, including multisite cohort, SCD global clinical trials, and SCD community engagement approaches. Trainees presented proposals for systematic literature reviews in key SCD research areas. This expert review emphasizes potential and prospects of SCDO-enabled data standards and harmonization to facilitate large-scale global SCD collaborative initiatives. As the fields of public and global health continue to broaden toward planetary health, the SCDO is well poised to play a prominent role to decipher SCD pathophysiology further, and co-design diagnostics and therapeutics innovation in the field.

## Introduction

Sickle Cell Disease (SCD) is one of the most common blood disorders and is caused by a single point mutation, which promotes sickling of erythrocytes due to polymerization of hemoglobin S. Each year, over 300,000 newborns are diagnosed with SCD globally and the incidence rates are expected to increase (Piel et al., 2013). The increase in SCD is compounded with its impact on morbidity and mortality rates. This has propelled SCD from a neglected disease to a recognized planetary health challenge (Ware, 2013). As the fields of public and global health continue to broaden toward planetary health, new approaches toward knowledge co-production and critical governance of medical innovations are urgently needed (Haines, 2017; Horton et al., 2014; Özdemir, 2019).

While traditional research efforts in SCD are commendable, with a few exceptions, many are conducted in silos (Makani et al., 2017), focus on limited populations, and in some instances suffer from statistical underpowering. To promote collaborative research and translation of research into health care interventions, an SCD ontology (SCDO) was created by a multidisciplinary working group (Group, 2019; Mulder et al., 2016).

The working group is made up of members of the H3AbioNet (Mulder et al., 2017), the Sickle Pan Africa Network (SPAN), and internationally recognized ontology experts. This working group currently operates under the auspice of the SickleInAfrica consortium (Makani et al., 2020). The SickleInAfrica consortium is made up of SPAN and two National Institutes of Health (United States)-funded projects: The Sickle Pan-African Consortium Network (SPARCo) and the Sickle Africa Data Coordinating Center (SADaCC) (Makani et al., 2020).

To our knowledge, the SCDO is the most comprehensive resource of standardized hierarchical descriptions of knowledge in the SCD domain and other related hemoglobinopathies (Group, 2019). Work is already underway to translate this resource into French and Portuguese and to create SCDO-framed data standards for SCD research. Such SCDO-driven data standards are expected to support reliable and robust exchanges and translation of knowledge to planetary health action in the field, not to mention more efficient data integration and interoperability from different and diverse sites for retrospective and prospective research.

Currently, the SCDO contains over 1575 standardized terms, which are categorized into 13 upper classes, which include phenotypes, genetic modifiers, guidelines, and quality of life and care, among others. Therefore, SCDO data standards associated data elements and data collection tools encompass multiple disciplines. The SCDO working group is continually updating the ontology, which is available on multiple platforms, including the project website (Sickle Cell Disease Ontology Working Group, 2017c), the European Bioinformatics Institute Ontology Look-up Service (Sickle Cell Disease Ontology Working Group, 2017b), Github (Sickle Cell Disease Ontology Working Group, 2017a), and Bioportal (Sickle Cell Disease Ontology Working Group, 2015).

This expert review presents the highlights and the lessons learned from the fourth SCDO workshop that marked the beginning of applications toward planetary health impact, and with an eye to empower and cultivate multisite SCD collaborative research.

## Context of the Fourth SCDO Workshop

The fourth SCDO workshop took place in November 2019 in Cape Town, South Africa and, as noted above, marked the beginning of the application phase of the ontology. The key aim of the workshop was to enable multisite SCD cohort collaborative research and clinical trials by using the SCDO to harmonize existing data elements from multiple sites. The structure of the SCDO is now stable enough to support research and translational work (Group, 2019). In fact, the SCDO framework has already been used to develop yet another disease-specific ontology, the Hearing Impairment Ontology (Hotchkiss et al., 2019).

**FIG. 1.**
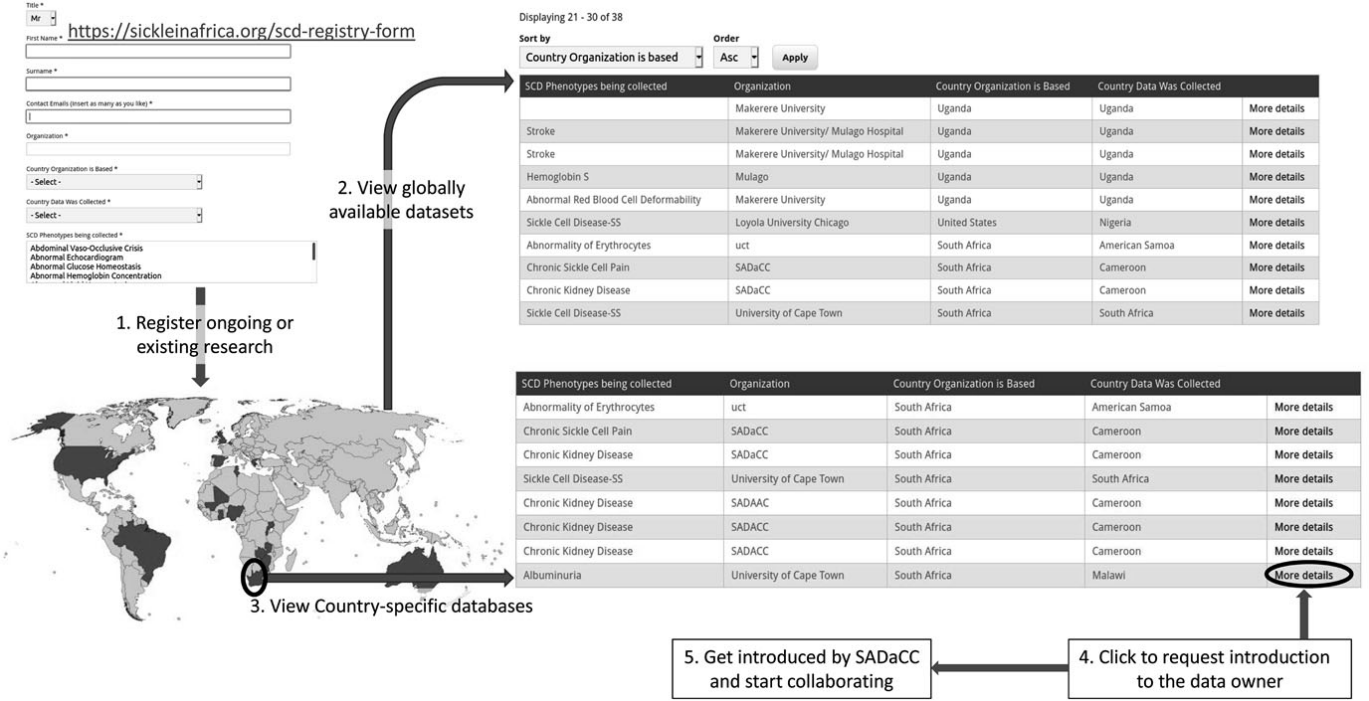
The process map to register, view, and access the Global SCD Registry Portal. SCD, sickle cell disease.

The workshop objectives included collecting data elements from the SCDO members who are actively engaged in data collection and research, in addition to demonstrating how data elements could be harmonized using the SCDO. An online searchable platform, the Global SCD Registry Portal, (https://www.sickleinafrica.org/registries-list) was developed to facilitate uploading of existing research data elements preworkshop. Other members of the SCDO working group who could not attend the workshop are likely to upload their data elements in anticipation of a global cohort study. The process for using this platform is shown in Figure 1. Additional objectives included developing participants' knowledge and skills for establishing cohort studies and clinical trials.

A total of 44 participants attended the meeting from 14 countries (Brazil, Cameroon, Ghana, Nigeria, Rwanda, Senegal, South Africa, Tanzania, Uganda, United Kingdom, United States of America, Malawi, Zambia, and Zimbabwe). Participants included experienced SCD researchers, junior researchers, and graduate students.

## Workshop Proceedings, Findings, and Outputs

The workshop provided an interdisciplinary global forum for participants to offer updates on SCD activities at their sites and report on interesting research outputs from some of their studies. An update on the SCDO was provided, followed by a session on data harmonization. Brainstorming sessions were held to develop new research ideas that use the harmonized SCD data or work across multiple sites, and discussions were held on how sites could get involved in clinical trials. The workshop included training activities in a variety of topics. An SCD patient advocate also presented at the workshop, giving a patient perspective on their aspirations, needs, and challenges.

### Sickle cell disease ontology

The SCDO was first released on BioPortal in 2017 and published in a peer-reviewed publication in 2019 (Group, 2019); however, the SCDO is in constant flux as new terms are added and the structure refined. During this fourth SCDO workshop, further additions and adjustments were made, particularly in the “Phenotype” class, where concepts of abnormality versus complications were separated. In addition, the mapping of SickleInAfrica data elements to the SCDO (for data standards) was started.

### Represented SCD networks and consortia

SPARCo aims to create a registry of 13,000 SCD patients across three countries, Tanzania, Nigeria, and Ghana, as a resource for future multisite cohort studies. All sites had achieved over 80% recruitment numbers of their targets. For example, Ghana had registered 2567 patients representing 85.6% of its target of 3000 patients. Various stakeholder engagement activities are ongoing, of particular note are the public-private partnerships in Ghana, aiming to make hydroxyurea widely accessible to SCD patients.

In addition, three sites are engaging and collaborating with government and advocacy organizations to ensure sustainability, delivering skills development activities for health care workers and researchers, developing standards of care for SCD patients at different levels of the health care systems, and developing cohort and implementation research proposals. Data from the three sites are collected with oversight from SADaCC and the SPARCo Hub. SADaCC is also responsible for data archiving.

The SickleGenAfrica network is funded by the National Institutes of Health (NIH) and is a collaborative project of genomics research coupled with capacity building (SickleGeneAfrica, 2020). SickleGenAfrica aims to recruit 7000 patients with SCD (children 1 year of age and older, and adults), who attend sickle cell clinics at six study sites in Ghana, Nigeria, and Tanzania. The main objectives include identifying genetic markers of cytoprotection proteins that neutralize hemolysis danger-associated molecular pattern molecules and acute organ damage.

In addition, the study aims to identify genome-wide determinants of malaria complications and echocardiographic abnormalities in patients with SCD. Ethics and community engagement are important components of the research. Ethics and psychosocial research possibilities were presented by SickleGenAfrica members during the workshop. A current survey being conducted within SickleGenAfrica was shown as an exemplar of ethics research in autonomy and decision making, and return of individual findings.

In addition, the European Union ARISE network highlighted their work packages and ongoing work in SCD research and training.

## Select Single-Country Studies

### Cameroon: genetic modifiers of the severity of SCD

Up to 1774 Cameroonian SCD patients were recruited in five regions of the country during two periods 2010–2013 and 2016–2018. The aim of the project was to explore the frequency and influence of genetic variants known to modulate the severity of SCD in Cameroon. Findings from the study were (1) the known fetal hemoglobin (HbF)-promoting loci (BCL11A and HBS1L-MYB) influence the expression of HbF in only 8.3% (Sickle Cell Disease Ontology Working Group, 2015) of Cameroonian patients, differing from the 20% to 50% observed in the African diaspora, (2) 36% of Cameroonian SCD patients have a deletion on the alpha thalassemia gene (Sickle Cell Disease Ontology Working Group, 2017a; Sickle Cell Disease Ontology Working Group, 2015), and (3) the predominant beta-globin gene haplotype is Benin (66%), followed by Cameroon (21%) (Sickle Cell Disease Ontology Working Group, 2017b). There was a high incidence of microalbuminuria (61%) and glomerular hyperfiltration (71%) in Cameroonian children and adolescents living with SCD.

The study found that clinical indicators tend to contribute highly to kidney phenotypic variations in SCD compared to the <15% variation explained by six kidney dysfunction-related genetic variants. There is need for further genetic and environmental research in this subpopulation.

### Evolution of the beta-hemoglobin gene cluster in Cameroonians

Given that SCD and malaria have co-evolved over the years, this study investigated the evolutionary selection pressure exacted by malaria on the structure of the beta-hemoglobin (*HBB*) gene cluster in Cameroonian individuals. Briefly, differences in the genetic distance of various Cameroonian ethnicities were observed, while longstanding knowledge of selection in the HBB gene cluster was confirmed. These findings can inform the design of future genetic association studies in specific African populations and guide the way inference is drawn from a few samples analyzed to the greater population.

### Brazil

#### The Recipient Epidemiology and Donor Evaluation Study-III Brazil SCD cohort

This is a large multicenter cohort established to characterize health outcomes in the Brazilian SCD population. Funded by the U.S. NIH, the cohort was enrolled between 2013 and 2015, with 2794 participants from four states (São Paulo, Rio de Janeiro, Minas Gerais, and Pernambuco). Key publications to date are as follows:

##### Clinical and genetic ancestry profile of a large multicenter SCD cohort in Brazil

A description of patient recruitment and study procedures, and baseline data collected during enrollment visits (2013–2015) (Carneiro-Proietti et al., 2018). The article also reports the sequencing of single nucleotide polymorphisms (SNPs) covering the entire genome.

##### Identification and characterization of hematopoietic stem cell transplantation candidates in an SCD cohort

This study found that 16% of children and 27% of adults had at least one indication for hematopoietic stem cell transplantation according to Ministry of Health criteria (Flor-Park et al., 2019). Two groups were compared, those with at least one indication and those without indications. The first group showed more severe disease with respect to clinical characteristics.

##### Clinical and genetic predictors of priapism in SCD (Cintho Ozahata et al., 2019)

This study describes a priapism prevalence of 14% in the cohort, with a higher occurrence in homozygous SS patients. Priapism was associated with avascular necrosis and pulmonary hypertension. A Genome-Wide Association Study (GWAS) also associated the risk of priapism with an SNP in the transforming growth factor beta-receptor gene. There are other articles in preparation, including quality of life in children and adults, profile of S-Beta0 and SB+ patients, pyrosequencing techniques for Hb mutations, RHD and RHCE variants, epidemiology of blood utilization and impact on chronic transfusion therapy and transfusion adverse events, risk and outcomes of HIV in SCD, and transfusion-transmitted infections in the cohort.

### Zambia: large-scale SCD point-of-care screening

In Zambia, a multidisciplinary network in a rural area has demonstrated the feasibility of using dental services to facilitate large-scale SCD point-of-care screening. Scaling up of dental services and other public health approaches, including the expanded program for immunization, has great potential for scaling up screening for SCD, including newborn screening in low-resource settings (Chunda-Liyoka et al., 2018).

### Zimbabwe: SCD registry

To understand the burden of SCD, the SCD phenotype in Zimbabwe, and gaps in the quality of care, a registry of children younger than 18 years was launched in 2018 at a tertiary level hospital in Harare. Preliminary data from the small cohort have shown that HbSS is the main genetic disease type. The SCD registry pilot was successful and will be continued; however, there is a need for data harmonization with other centers in Africa to allow collaborative work and data comparability. Additional studies are required to further characterize the SCD genotype and phenotype and establish newborn screening, and to provide data for policy makers and allow proper health care planning for patients with SCD in Zimbabwe.

### Harmonization of SCD data elements

The SickleInAfrica data elements are maintained on the REDCap platform. Following workshop sessions about REDCap (basics, data elements, and data harmonization) using the SCDO framework, participants highlighted what they considered the essential data elements in their SCD registries. A consensus was reached and the SickleInAfrica data elements adjusted accordingly.

A live hands-on session on harmonization of new datasets was conducted for participants maintaining registries or collecting data for SCD research. SADaCC researchers had developed a spreadsheet that auto-identifies common data elements and Python scripts that carry out the harmonization. Participants provided their feedback on the experience and challenges faced during this harmonization process, which will be used to refine this process.

#### Training

##### Data quality and assurance processes

Participants received training on how REDCap features can be utilized to improve data quality during patient recruitment. Participants were introduced to R packages, which can calculate and summarize data quality-relevant information for a specified dataset.

##### Big data analytics

A video presentation was delivered by one of the co-leads of the Big Data Analytics course, which was piloted to SickleInAfrica researchers earlier in 2019. Background on the use of Big Data in SCD was given and the different training modules were also described.

##### Ethics, legal and social implications

The ethics, legal and social implications (ELSI) framework developed to support the SPARCo registry and SickleInAfrica data governance issues among other items was finalized during this meeting by all participants. This included the data sharing and authorship policies and consent/assent forms. Details of the processes followed in developing the ELSI framework have been published in a separate article (Munung et al., 2019). The consortium agreed to develop ELSI research questions, which could answer important questions for the implementation and application of the SCDO for research purposes.

It was agreed that patient engagement should be a core activity of the projects to maximize the benefits of the SCDO to patient advocacy groups. The SCDO is currently being translated into layperson language to allow patient groups to benefit from this resource. All SickleInAfrica sites have extensive experience working with local SCD patient groups; however, proper engagement models are lacking. It was agreed upon to work together with patient representatives across sites to design a patient engagement framework that is going to be used as a model within SickleInAfrica.

The SCDO Quality-of-Life and Care definitions were utilized to encourage workshop participants to interrogate their retrospective data, identifying possible quality-of-life indicators (e.g., pain, physical functioning, social functioning, education, and occupation) and possible calculation of disability-adjusted life year. For prospective studies, participants were presented with quality-of-life and care issues such as validation of measures, literacy, language differences, and consideration of appropriate pain, quality of life (e.g., EQ-5D—available in some African languages), health utility indices, and quality-adjusted life year assessments defined in the SCDO.

## Dynamic Development of Literature Reviews

Fifteen trainees from the pilot SickleInAfrica Big Data Analytics training were taught how to formulate research questions for literature reviews focusing on the SCD. This training included an introductory lecture on SCD. After the training, fellows presented their literature review concepts. To attend the next SickleInAfrica consortium meeting, trainees would need to show substantial progress in the development of their literature reviews with plans to publish the article. The need for advanced training and expansion of this training to include proposal development and scientific writing, which is scheduled for the near future, was highlighted. Challenges in the development of literature reviews included lack of time and limited access to journals.

## Collaborative Grant Writing and Funding Opportunities

To support future collaborations among participants, training was provided in collaborative grant writing. The differences between writing collaborative grant proposals compared to single group proposals were highlighted. Key skills identified were leadership, communication, and planning. The collaborative process involves orientation, conflict, and emergence and reinforcement phases (Dopke and Crawley, 2013). The understanding of Belbin team roles and their strengths and weaknesses were proposed to facilitate efficient teamwork. Using SCD as an example, information on the availability and accessibility of grant funding opportunities for groups in low- and middle-income countries was provided.

## Multisite Study Design

This session described observational studies, distinguishing cross-sectional studies, case–control studies, and cohort studies. Cohort studies were further distinguished as follows: concurrent prospective, nonconcurrent prospective, cross-sectional prospective, truly retrospective, and cross-sectional retrospective. The session suggested that the scope of observational studies in SickleInAfrica will mostly include cross-sectional studies, retrospective studies, and prospective nonconcurrent studies.

The core of the session focused on potential challenges that could be encountered analyzing SickleInAfrica data, which include reassessing the distribution of diseases and health statuses using available data, specifically:

- representativeness of the study populations
- the accuracy of diagnosis and definition of health statuses
- completeness of information on diseases and health statuses (missing data).

Due to variation across sites, analysis of SickleInAfrica multicenter observational studies is likely to require extensive data quality checks. Possible solutions to some of these challenges were discussed, including imputation of missing data and employing individual participant data meta-analysis for multicenter observational studies. Examples were used to illustrate challenges and how they have been addressed in published studies such as the Asia Pacific Cohort Studies collaboration (Kengne et al., 2009) (Asia Pacific Cohort Studies Collaboration et al., 2007), the Health Surveys of England cohorts (Kengne et al., 2012), the pan-European EPIC-InterAct study (Kengne et al., 2014), and the Demographic Health Survey data (Caleyachetty et al., 2014).

## Basics of Local and Global Clinical Trial Design

A representative from the African Academy of Sciences (AAS) presented a prerecorded video talk on the current status, new priorities, and new opportunities for clinical trials in Africa. AAS aims to create a platform to support communities of clinical trials. Subsequently, the main features and principles of clinical trials were discussed.

A strong emphasis was placed on conducting research in an ethical manner. Recent clinical trial examples (two from Europe and one from Africa) illustrated how quickly sidestepping or ignoring safeguards may lead to detrimental patient outcomes. The discussion was particularly timely, given the announcement a week earlier of a new major collaborative effort between the NIH and the Bill and Melinda Gates Foundation to develop advanced therapies for SCD (“NIH launches new collaboration to develop gene-based cures for sickle cell disease and HIV on global scale | National Institutes of Health [NIH],” 2020), an effort that will require numerous clinical trials of novel therapies both in Africa and overseas.

In addition, the difference between early-phase and late-phase trials was explained, with the double-blinded, placebo-controlled, randomized controlled trial showcased as the (often infeasible) gold standard for the latter. The challenges in generalizing trial results to entire patient populations, when the trials are usually carried out on convenience samples, were also discussed versus case studies. Recent clinical trials with severe ethical transgressions leading to injury or death were highlighted, including “Abdullahi vs. Pfizer,” or “TeGenero,” or “BIA 10-2474.”

The AAS representative led a discussion on how a community of SCD stakeholders could be created to support clinical trials across the continent (within the AAS Clinical Trials Community platform currently under development). Therefore, in addition to the clinical trials proposed in Table 2, the group also highlighted suggestions and their needs to the AAS.

Integration of biomarker candidates in global clinical trials can help not only personalized medicine in the clinic but also inform broader generalization of trial results across world populations in the future (Şardaş and Kendirci, 2019).

## Designing and Analyzing SCD Existing Data

Basics of designing observational studies based on existing data were introduced. Participants were encouraged to collaborate with a quantitative analyst starting in the conceptualization and design stages rather than only for the analysis stage. Potential biases and limitations of observational studies, including confounding variables and other causal relationships, were demonstrated. The need to strike a balance between *a priori* analysis plans and the process of discovery was discussed, as well as the importance of high-quality descriptive work, besides embarking upon more sophisticated model-based analysis. Some case studies were provided as examples for observational and clinical trial talks and are summarized in Table 1.

**Table 1.** Sickle Cell Disease Ontology Workshop Case Studies from Multisite Retrospective Clinical Trials and Study Design Talks

Type of study	Summary	References
Multisite observational	Cystic fibrosis study demonstrating the power of “simple” descriptive analysis	Cogen et al. (2017)
Multisite observational	Prevalence of Behavioral Risk Factors for Cardiovascular Disease in Adolescents in Low-Income and Middle-Income Countries: An Individual Participant Data Meta-Analysis	Caleyachetty et al. (2015)
Multiste observational	Tobacco use in pregnant women: Analysis of data from Demographic and Health Surveys from 54 low-income and middle-income countries	Caleyachetty et al. (2014)
Multisite observational	Noninvasive Risk Scores for Prediction of Type 2 Diabetes (EPIC-InterAct): A Validation of Existing Models	Kengne et al. (2014)
Multisite observational	Systolic Blood Pressure, Diabetes and the Risk of Cardiovascular Diseases in the Asia-Pacific Region	Asia Pacific Cohort Studies Collaboration et al. (2007)
Multisite prospective	Association of C-reactive protein with cardiovascular disease mortality according to diabetes status: pooled analyses of 25,979 participants from four U.K. Prospective Cohort Studies	Kengne et al. (2012)
Single-site observational study	Anemia management among dialysis patients, showing a complicated causal relationship between two variables	Pollack et al. (2018)
Multisite observational	Combining different syndrome groups in a questionable manner, but the outcome still contributed to scientific knowledge	Salehi et al. (2018)
Randomized clinical trial	Randomized clinical trial in India for high-dose Vitamin A and deworming. The lack of placebo was used to attack it, but possibly most attackers just did not like its Null-effect outcome	Awasthi et al. (2013)
Double-blind randomized control trial	MORDOR trial for azithromycin prophylaxis in African children. Large, textbook-quality placebo-controlled, double-blind RCT, which is nevertheless controversial because it is unclear how to generalize from the somewhat disparate results in the trial's 3 sites. As a way out, additional, even larger trials are planned	Keenan et al. (2018)

### Multisite collaborative study proposals

In two sessions, participants developed detailed concepts for future studies. For the observational studies, two studies were proposed, a descriptive study and a genetic study. The descriptive study will focus on a meta-analysis across all the SPAN countries and would include physical attributes, disease, and phenotypes of patients. The genetic study will be a GWAS meta-analysis of HbF based on existing GWAS data from Tanzania, Cameroon, Brazil, and United States. In addition, research questions will be developed by the Big Data Analytics Trainees. Groups were encouraged to align their clinical trials to the SPARCo research focus areas; a summary of the proposed clinical trial studies is in Table 2.

**Table 2.** Proposed Clinical Trials in Sickle Cell Disease

Area	Trials
Prevention of pneumococcal infections in SCD	Alternatives to penicillin V prophylaxis
	Pneumococcal vaccination booster schedules
	Education and awareness
	Micronutrient supplementation
Newborn screening	Cost-effective methods for delivering newborn screening for SCD
Hydroxyurea	Hydroxyurea dosage in adults with SCD: Fixed low-dose versus maximum tolerated dose
Nutrition	Intervention for poor growth in infants and young children with SCD
Malaria and SCD	Chemoprophylaxis regimens with or without insecticide-treated bednets

SCD, sickle cell disease.

## Planetary Health Solutions and Research in SCD prioritized

The SCD research field has historically been trivialized for scientific and technological advancements due to a range of inherent biases that exist in common global frameworks. Planetary health is a public health discipline established in 2014, calls for critical governance and implementation frameworks that if adopted for the SCD research field could provide effective planetary health solutions that consider and encompass natural systems upon which human health depends (Haines, 2017; Horton et al., 2014; Whitmee et al., 2015).

A unique differentiating feature of planetary health is that not only is its scope broad but it is also critically informed, for example, by emphasizing and formally recognizing the importance of ecological and political determinants of health. Ecological determinants include, for example, pandemics such as COVID-19, whereas political determinants include power asymmetries and historical and modern-day social injustices in science and quotidian life that are often overlooked and yet markedly impact the health of humans and planetary ecosystems.

To these ends, this report demonstrates a shift in the SCD field toward participatory research, highlighting the relevancy of a recently proposed planetary health knowledge critical governance framework to advocate for innovative and more participatory and inclusive approaches in global science and research (Özdemir, 2019).

In addition, the SCDO has potential to enable tailoring of planetary biomedical and clinical solutions through promotion of human collaborative research across biological, social, ecological, and political determinants of health. For example, the SCDO could enable implementation of Panvigilance, a systems pharmacovigilance planetary approach, which holds great promise toward enhanced management of adverse drug reactions, drug safety, and efficacy. Panvigilance integrates biomarkers from edge populations in clinical trial designs for improved detection of unintended drug effects, whether adverse, toxic, or therapeutic (Şardaş and Kendirci, 2019).

## Conclusions

The SCDO working group agreed on deliverables to foster collaborative research, strengthening research quality and scientific writing skills of the participants. Most importantly, the workshop demonstrated the potential of SCDO-enabled data standardization to enable large-scale global SCD collaborative initiatives using existing disparate multisite datasets. While biomedical science and clinical applications broaden toward planetary health, the SCDO is well poised to play a prominent role to decipher SCD pathophysiology further, and co-design diagnostics and therapeutics innovation in the field.

## Abbreviations Used

AAS: African Academy of Sciences
DAMP: danger associated molecular pattern
ELSI: ethics, legal and social implications
HBB: beta-hemoglobin
HbF: fetal hemoglobin
NIH: National Institutes of Health
SCD: sickle cell disease
SCDO: sickle cell disease ontology
SADaCC: Sickle Africa Data Coordinating Center
SPAN: Sickle Pan Africa Network
SPARCo: Sickle Pan-African Consortium Network
